# *Cis*‐2‐decenoic acid modulates *Pseudomonas aeruginosa* virulence through a noncanonical transcriptional regulator

**DOI:** 10.1002/mlf2.70044

**Published:** 2025-12-25

**Authors:** Shihao Song, Jingyun Liu, Bing Wang, Yang Si, Hongguang Han, Xiuyun Sun, Mingfang Wang, Binbin Cui, Guangliang Wu, Yongliang Huo, Liangxiong Xu, Beile Gao, Liang Yang, Xiaoxue Wang, Lian‐Hui Zhang, Yinyue Deng

**Affiliations:** ^1^ Key Laboratory of Tropical Biological Resources of Ministry of Education, School of Pharmaceutical Sciences Hainan University Haikou China; ^2^ Integrative Microbiology Research Center South China Agricultural University Guangzhou China; ^3^ School of Pharmaceutical Sciences (Shenzhen) Shenzhen Campus of Sun Yat‐sen University, Sun Yat‐sen University Shenzhen China; ^4^ Zhengzhou Health College Zhengzhou China; ^5^ Department of Microbiology, College of Life Sciences Nanjing Agricultural University Nanjing China; ^6^ Pharmacy Department The Affiliated Lihuili Hospital of Ningbo University Ningbo China; ^7^ Experimental Animal Center Guangzhou Medical University, Guangzhou China; ^8^ School of Life Sciences Huizhou University Huizhou China; ^9^ Guangdong Key Laboratory of Marine Materia Medica South China Sea Institute of Oceanology, Chinese Academy of Sciences Guangzhou China; ^10^ School of Medicine Southern University of Science and Technology Shenzhen China

**Keywords:** *cis*‐2‐decenoic acid, fatty acid‐CoA ligase, *Pseudomonas aeruginosa*, quorum sensing, virulence

## Abstract

Diffusible signal factor (DSF)‐family quorum sensing (QS) signals are widely utilized by many pathogenic bacteria to modulate various biological functions and virulence. Previous studies showed that *cis*‐2‐decenoic acid (*cis*‐DA) is involved in the modulation of biofilm dispersion in *Pseudomonas aeruginosa*, but the regulatory mechanism is unclear. Here, we report that *cis*‐DA regulates the physiology and virulence of *P. aeruginosa* through FadD1, a long‐chain fatty acid‐CoA ligase. *cis*‐DA specifically binds to FadD1 and enhances the binding ability of FadD1 to the target gene promoter DNA regions. Further analysis showed that FadD1 is a global regulatory factor that controls the transcription of various target genes. Moreover, FadD1 showed catalytic activity on *cis*‐2‐dodecenoic acid (BDSF) of *Burkholderia cenocepacia* and enhanced the competitiveness of *P. aeruginosa*. Together, our work presents a new DSF‐type QS signaling system in *P. aeruginosa*, which is highlighted by the signal receptor evolved from a canonical enzyme of fatty acid metabolism.

## Impact statement

Quorum sensing (QS) is widely presented as an intercellular communication mechanism in bacteria. This study demonstrates that FadD1, a long‐chain fatty acid‐CoA ligase with a DNA‐binding leucine zipper motif, is a receptor of *cis*‐2‐decenoic acid (*cis*‐DA) in *Pseudomonas aeruginosa*. Perception of *cis*‐DA enhances the binding of FadD1 to the *lasR* promoter to control the *rhl*‐ and *pqs*‐QS systems. This study reveals a class of specific fatty acid‐CoA ligases with a leucine zipper motif as novel QS signal receptors, playing important roles in the virulence of bacteria.

## INTRODUCTION

Quorum sensing (QS) is a cell‐to‐cell communication mechanism utilized by numerous species of bacteria. The acyl homoserine lactone (AHL) produced by *Vibrio fischeri* is the first QS signal discovered in Gram‐negative bacteria^1^, ^2^, ^3^, ^4^, ^5^, and many other types of QS signals have now been discovered^6^, ^7^, ^8^, ^9^, ^10^, ^11^, ^12^, ^13^, ^14^, ^15^, ^16^, including the diffusible signal factor (DSF)‐family signals, such as *cis*‐11‐methyl‐dodecenoic acid (DSF) and *cis‐*2‐dodecenoic acid (BDSF). *Pseudomonas aeruginosa* is the main source of opportunistic infections in hospitals and has evolved multiple types of QS systems, such as *las*, *pqs*, and *rhl* ^17^, ^18^, ^19^. The *las* and *rhl* AHL QS systems use *N*‐3‐oxo‐dodecanoyl‐l‐homoserine lactone (3‐oxo‐C12‐HSL) and *N*‐butyryl‐l‐homoserine lactone (C4‐HSL), respectively, to regulate the biological functions, like biofilm formation, motility, and virulence^20^, ^21^, ^22^, ^23^, ^24^. The *pqs* system uses 2‐heptyl‐3‐hydroxy‐4(1H)‐quinolone (PQS) to regulate physiology and pathogenesis^25^, ^26^, ^27^, ^28^, ^29^. These three QS systems are hierarchically interrelated, and the *las* system was confirmed to control both the *rhl* and *pqs* systems^17^, ^30^. Moreover, the DSF‐type QS signal, *cis*‐2‐decenoic acid (*cis*‐DA), was also revealed to serve important functions in *P. aeruginosa* ^31^. The production of *cis*‐DA needs the enoyl‐CoA synthase DspI^32^. The inactivation of DspI leads to a significant reduction in biofilm dispersion^33^, ^34^. However, it is not yet clear how *cis*‐DA regulates biofilm dispersion, and its receptor and downstream signaling network remain to be investigated.

Fatty acid‐CoA synthetases (FACSs), widely present in organisms, are a class of enzymes that activate alkanoic acids to CoA esters^35^ and show broad substrate specificity^36^. FadD, a long‐chain fatty acid‐CoA ligase, is responsible for activating endogenous long‐chain fatty acids into acyl‐CoAs^37^. RpfB was reported as a long‐chain fatty acid‐CoA ligase in *Xanthomonas campestris* pv. *campestris* (*Xcc*); it is involved in the activation of various fatty acids into CoA esters in vitro^38^, ^39^. Intriguingly, RpfB is required for the turnover of both DSF and BDSF QS signals in vivo^39^. The *rpfB* deficiency mutant increases the levels of both DSF and BDSF signals and displays increased virulence in *Xcc* ^40^, ^41^. Moreover, the RpfB‐dependent QS signal conversion has also been detected in various bacterial species^40^.

Transcription factors (TFs) can bind to specific DNA sequences and control gene transcription initiation activity^42^. Once TFs bind to specific DNA, they can promote or block the recruitment of RNA polymerase to control transcriptional expression^43^. Although there are significant differences in the structures and functions of TFs, some polypeptide motifs can bind to the main slot of DNA, including helix–turn–helix (HTH), helix–loop–helix (HLH), zinc fingers, and leucine zippers^44^, ^45^, ^46^, ^47^.

In this study, we found that FadD1 of *P. aeruginosa* could convert BDSF into *cis*‐2‐dodecenoic acid‐CoA and reduce the competitiveness of *Burkholderia cenocepacia*. Intriguingly, FadD1 serves an additional function as a global transcriptional regulator in *P. aeruginosa*, regulating the QS hierarchy and the expression of various target genes by sensing *cis*‐DA, and then modulates the biological functions and virulence. These findings indicate that FadD1 is a member of a novel class of QS signal receptors that control the important physiology and virulence of bacterial pathogens, with an additional role in fatty acid oxidation to catalyze the formation of fatty acyl‐CoA.

## RESULTS

### FadD1 of *P. aeruginosa* shows enzyme activity on the BDSF signal

**Figure 1:**
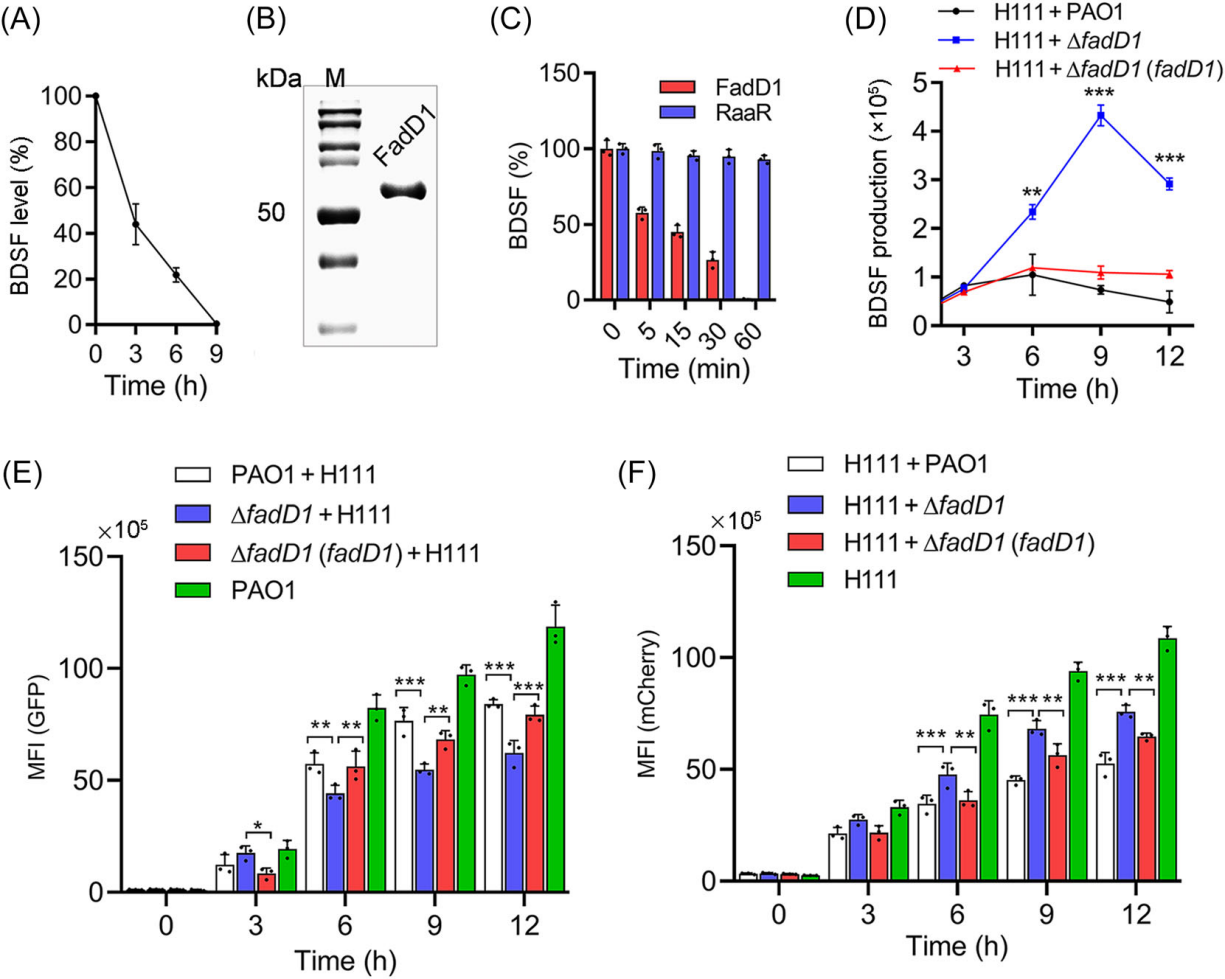
Effect of *fadD1* on the competitive capability of *Pseudomonas aeruginosa* against *Burkholderia cenocepacia*. (A) Analysis of the *cis*‐2‐dodecenoic acid (BDSF) signal quenching ability of *P. aeruginosa* PAO1. (B) Sodium dodecyl sulfate – polyacrylamide gel electrophoresis (SDS‐PAGE) of the purified FadD1 protein. (C) Analysis of the enzymatic quenching activity of FadD1 on the BDSF signal. A non‐BDSF‐degrading protein RaaR, the anthranilic acid receptor protein of *Ralstonia solanacearum*, was used as a control. (D) BDSF signal production of the *B. cenocepacia* H111 strain cocultured with *P. aeruginosa* PAO1 wild‐type, ∆*fadD1,* and ∆*fadD1*(*fadD1*) strains. (E) Green mean fluorescence intensity (MFI) of the *P. aeruginosa* PAO1 wild‐type, ∆*fadD1,* and ∆*fadD1*(*fadD1*) strains carrying the GFP expression vector. (F) Red MFI of the *B. cenocepacia* H111 strain carrying the mCherry fluorescent protein expression vector. The data are the means ± standard deviations of three independent experiments. The *P. aeruginosa* PAO1 wild‐type, Δ*fadD1,* and ∆*fadD1*(*fadD1*) strains carried the GFP expression vector. The *B. cenocepacia* H111 strain carried the mCherry fluorescent protein expression vector. The *P. aeruginosa* PAO1 wild‐type, ∆*fadD1,* and ∆*fadD1*(*fadD1*) strains were cocultured with *the B. cenocepacia* H111 strain at a ratio of 1:4 (v/v) at OD_600_ of 0.1. The statistical comparisons were performed using two‐way ANOVA (*p < 0.05; **p < 0.01; and ***p < 0.001).

BDSF was first found to regulate the biofilm formation, motility, and virulence of *B. cenocepacia* ^9^, ^10^, ^11^, ^12^, ^48^, ^49^. It was subsequently reported that the BDSF from *B. cenocepacia* interferes with the type III secretion system (T3SS) and QS systems of *P. aeruginosa* at a concentration as high as 0.25 mM^50^. Interestingly, we found that BDSF could also be degraded when it was added to the culture of *P. aeruginosa* PAO1. The BDSF content in the culture of *P. aeruginosa* decreased to 21.78% after 6 h of incubation (Figure 1A). Previous studies demonstrated that RpfB is a long‐chain fatty acid‐CoA ligase responsible for BDSF and DSF signal turnover in *Xcc* ^38^, ^39^. To discover the enzyme that degrades BDSF, we then searched RpfB homologs in the genome of *P. aeruginosa* PAO1 by using the Basic Local Alignment Search Tool (BLAST) algorithm^51^. We found six RpfB homologs, FadD1‐6^52^; among them, FadD1 showed the highest homology, with 55.35% identity with RpfB. FadD1 contains 562 aa with a predicted molecular weight of 61.67 kDa, purified to homogeneity using affinity chromatography (Figure 1B). We also purified a non‐BDSF‐degrading protein RaaR, anthranilic acid receptor protein of *Ralstonia solanacearum* ^53^, as a control (Figure S1). The in vitro enzymatic activity assays showed that when FadD1 was mixed with BDSF, the free BDSF level in the mixture decreased to nearly undetectable levels after 1 h (Figure 1C), but RaaR displayed no degradation activity. These results suggested that FadD1 shows enzyme activity on BDSF. Also, deletion of *fadD1* in *P. aeruginosa* did not affect its growth rate (Figure S2).

We then investigated the in vivo activity of FadD1 in *B. cenocepacia* H111 by overexpressing the *fadD1* gene. Overexpression of *fadD1* did not affect its growth rate but markedly reduced BDSF production of the bacterial cells (Figures S3A,B). RpfF_Bc_ is required for BDSF synthesis in *B. cenocepacia* H111, and as expected, deletion of *rpfF*_*Bc*_ resulted in complete abolishment of the production of BDSF (Figure S3B). Interestingly, overexpression of *fadD1* in the *B. cenocepacia* H111 strain reduced the biofilm formation, swarming motility, and protease activity, which could be restored by adding 5 µM exogenous BDSF (Figure S3C–E). Overexpression of *fadD1* in the wild‐type strain H111 also decreased cytotoxicity (Figure S3F).

### FadD1 boosts the competitiveness of *P. aeruginosa*

As the communication between *B. cenocepacia* and *P. aeruginosa* could be mediated by BDSF^50^ and FadD1 shows catalysis activity on BDSF, we investigated whether FadD1 plays a role in the competitive interactions between these two bacterial species. To this end, we cocultured the green fluorescent protein (GFP) fluorescence‐labeled *P. aeruginosa* wild‐type, ∆*fadD1*, and complemented strains in the presence of *B. cenocepacia* H111 strain labeled with mCherry and measured the BDSF production of *B. cenocepacia* (Figure 1D) and the mean fluorescence intensity (MFI) of both *P. aeruginosa* and *B. cenocepacia* at different time points (Figure 1E,F). The results demonstrated that the absence of *fadD1* led to a sharp decrease in the degradation ability of *P. aeruginosa* toward the BDSF signal. The BDSF signal yield of *B. cenocepacia* cocultured with the *P. aeruginosa* ∆*fadD1* mutant strain was more than five times higher than that when it was cocultured with *P. aeruginosa* wild‐type or ∆*fadD1*(*fadD1*) complemented strains at 9 h postinoculation (Figure 1D). On the other hand, we found that the GFP MFI of the *P. aeruginosa* ∆*fadD1* strain was lower than that of *P. aeruginosa* wild‐type and ∆*fadD1*(*fadD1*) complemented strains when they were cocultured with the *B. cenocepacia* H111 strain at 6, 9, and 12 h (Figure 1E). The mCherry MFI of *B. cenocepacia* cocultured with *P. aeruginosa* ∆*fadD1* was higher than that of *B. cenocepacia* cocultured with *P. aeruginosa* wild‐type and complemented strains (Figure 1F). The results demonstrated that FadD1 plays an important role in the competition between *P. aeruginosa* and *B. cenocepacia*.

### Mutation of FadD1 impairs the biological function and pathogenicity of *P. aeruginosa*

**Figure 2:**
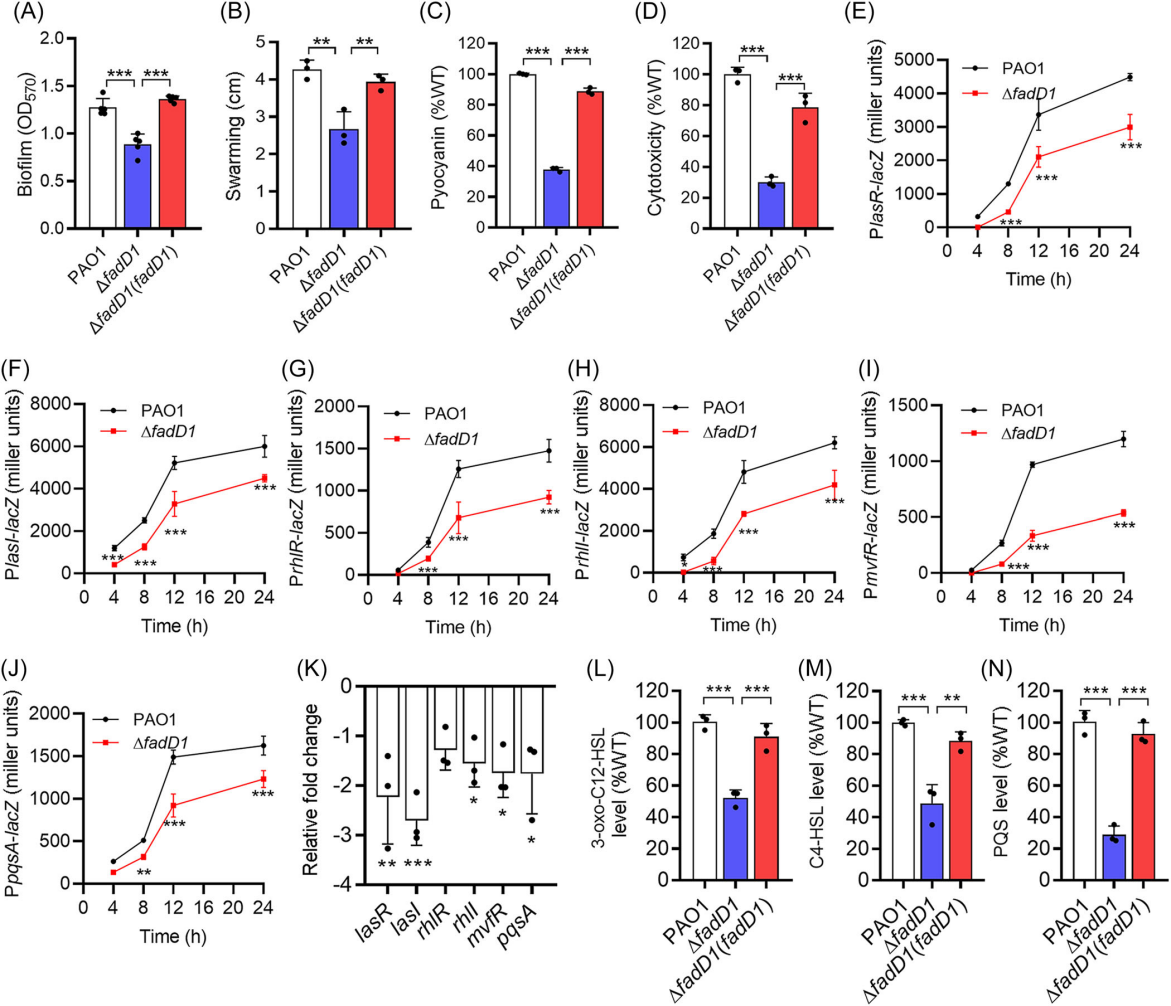
Effects of *fadD1* on the QS‐regulated phenotypes and QS of *P. aeruginosa*. (A–D) Analysis of biofilm formation (A), swarming motility (B), pyocyanin production (C), and virulence (D) in *P. aeruginosa* PAO1. (E–K) Effects of *fadD1* on the gene expression levels of *lasR* (E), *lasI* (F), *rhlR* (G), *rhlI* (H), *mvfR* (I), and *pqsA* (J) by assessing the β‐galactosidase activity of the promoter–*lacZ* transcriptional fusions and by RT‐qPCR (K). (L–N) Production of 3‐oxo‐C12‐HSL (L), C4‐HSL (M), and PQS (N) in the *P. aeruginosa* PAO1 wild‐type, ∆*fadD1,* and ∆*fadD1*(*fadD1*) strains at an OD_600_ of 3.0. The production of QS signals in the *P. aeruginosa* PAO1 wild‐type strain was arbitrarily defined as 100%. The data are means ± standard deviations of three independent experiments. The statistical comparisons were performed using one‐way ANOVA or two‐way ANOVA (one‐way ANOVA for (A), (B), (C), (D), (L), (M), and (N); two‐way ANOVA for (E), (F), (G), (H), (I), and (J), two‐way ANOVA; *p < 0.05; **p < 0.01; and ***p < 0.001).

Since FadD1 plays an important role in the competition between *P. aeruginosa* and *B. cenocepacia*, we continued to investigate whether the FadD1 plays a key role in regulating the biological functions and virulence. We found that the absence of *fadD1* resulted in 30.47%, 36.72%, and 62.15% reduction in biofilm formation, swarming, and pyocyanin production, respectively (Figure 2A–C). Deletion of *fadD1* also reduced the cytotoxicity of *P. aeruginosa* to A549 cells by 69.78% at 8 h postinoculation (Figure 2D). We also tested whether there was a change in the expression levels of upstream (*PA3298*) and downstream (*fadD2*) genes of the *fadD1* mutant gene. The results indicated that there was no change in the expression of upstream and downstream genes in the ∆*fadD1* strain (Figure S4). To further investigate the regulatory roles of FadD1, we used RNA sequencing (RNA‐Seq) to analyze and compare the transcriptomes of wild‐type and ∆*fadD1* strains. The results indicated that the expression levels of 208 genes decreased and those of 23 genes increased in the ∆*fadD1* strain (|log_2_ fold‐change|≥ 1.0) (Table S1 and Figure S5A). These differentially expressed genes are associated with many biological functions (Table S1 and Figure S5B). These genes include the *las*, *rhl*, and *pqs* QS system genes *PA1432* (*lasI*), *PA1430* (*lasR*), *PA3476* (*rhlI*), *PA3477* (*rhlR*), *PA0996* (*pqsA*), and *PA1003* (*mvfR*), which were shown to be associated with the pathogenicity of *P. aeruginosa*.

### FadD1 modulates the QS systems of *P. aeruginosa*

As FadD1 affects the QS‐regulated genes and phenotypes of *P. aeruginosa* (Figures 2A–D and S5 and Table S1), we inferred that FadD1 might affect the QS systems. Analysis of the expression profiles of *lacZ* under the control of the *lasR*, *lasI*, *rhlR*, *rhlI*, *mvfR,* and *pqsA* promoters showed that the absence of *fadD1* led to a decrease in the expression levels of *lasR*, *lasI*, *rhlR*, *rhlI*, *mvfR,* and *pqsA* (Figure 2E–J), which are signal receptor genes and synthase‐encoding genes of *las*, *rhl,* and *pqs* systems. RT‐qPCR analysis and RNA‐seq indicated that the deletion of *fadD1* led to decreased expression levels of *lasR*, *lasI*, *rhlR*, *rhlI*, *mvfR,* and *pqsA* (Figure 2K). Then, we compared the yields of 3‐oxo‐C12‐HSL, C4‐HSL, and PQS in the wild‐type, *fadD1* mutant, and complemented strains. We found that the yields of 3‐oxo‐C12‐HSL, C4‐HSL, and PQS were reduced in the ∆*fadD1* strain by 47.8%, 51.13%, and 71.13%, respectively (Figure 2L–N). The concentrations of 3‐oxo‐C12‐HSL, C4‐HSL, and PQS of the *fadD1* mutant strain were 0.59, 0.37, and 0.9 μM, respectively. All the results indicated that FadD1 positively regulated the QS systems of *P. aeruginosa*.

### FadD1 directly binds to the promoter of multiple genes to regulate the target gene expression

**Figure 3:**
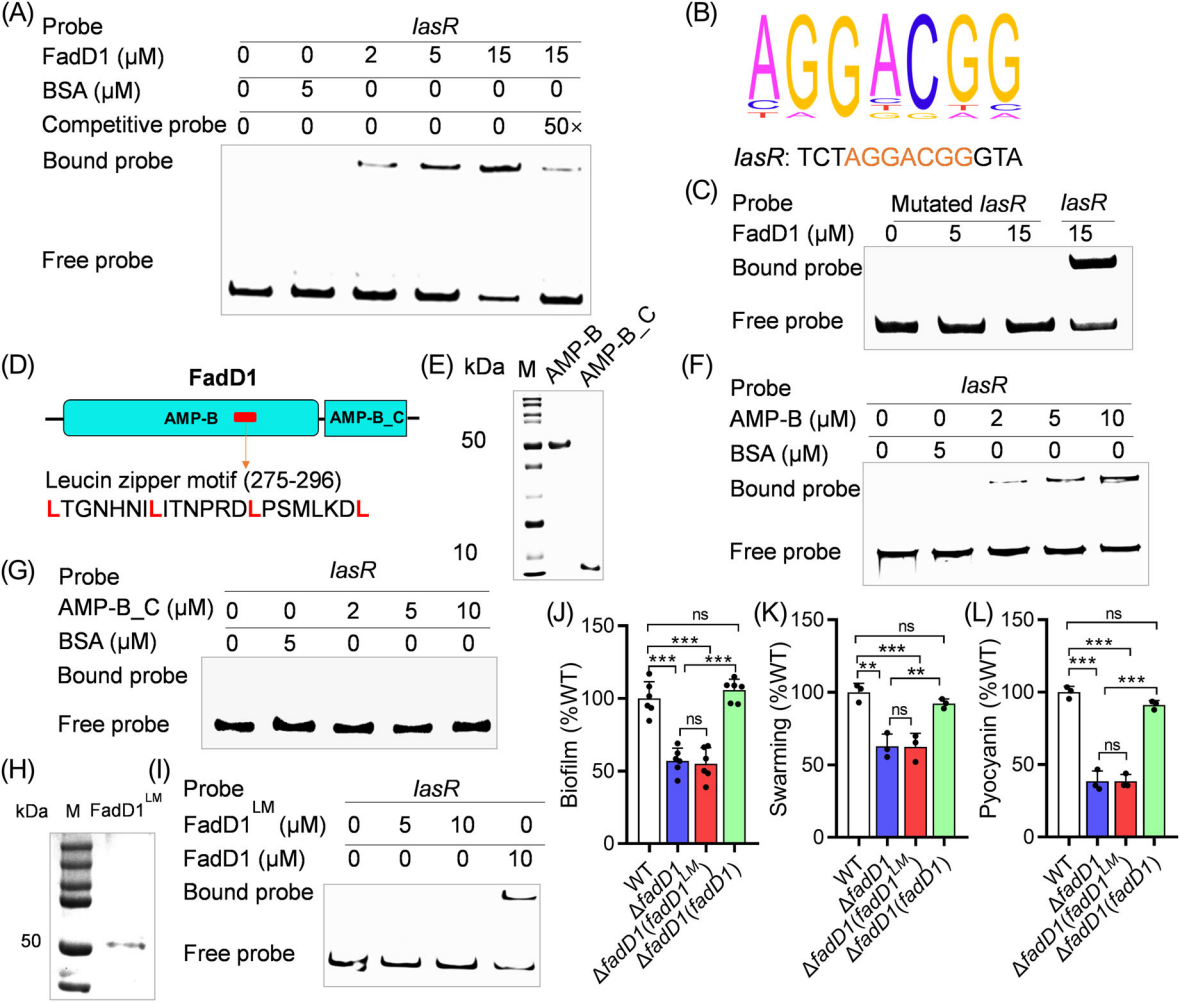
Analysis of the binding of FadD1 to target gene promoters. (A) Electrophoretic mobility shift assay (EMSA) analysis of in vitro binding of FadD1 to the promoter of *lasR*, in which the biotin‐labeled 339‐bp *lasR* promoter DNA probe was used for the protein‐binding assay. A protein–DNA complex, represented by a band shift, was formed when different concentrations of the FadD1 protein were incubated with the probe at room temperature for 30 min. (B) A potential FadD1‐binding region identified by ChIP‐seq. The conserved sequence is shown in orange. (C) Analysis of the binding between FadD1 and the mutated *lasR* promoter with deletion of the FadD1‐binding sequence AGGACGG. EMSA analysis was performed in vitro. (D) Domain structure analysis of FadD1 (https://www.ebi.ac.uk/Tools/hmmer/). The red box represents the leucine zipper motif (https://www.novopro.cn/tools/motifscan.html) in the AMP‐B domain. (E) SDS‐PAGE of the purified AMP‐B domain and the AMP‐B_C domain of the FadD1 protein. (F, G) EMSA analysis of in vitro binding of the AMP‐B domain (F) and the AMP‐B_C domain (G) to the promoter of *lasR*. (H) SDS‐PAGE of the purified mutated FadD1 protein (FadD1^LM^: L275A L282A L289A L296A). (I) EMSA analysis of the in vitro binding of the mutated FadD1 protein to the promoter of *lasR*. *In trans* expression of the mutated *fadD1* could not restore biofilm formation (J), swarming motility (K), and pyocyanin production (L) in the *P. aeruginosa* Δ*fadD1* strain. The EMSA experiments were performed three times, and representative images from one experiment are shown. The data are the means ± standard deviations of three independent experiments. The statistical comparisons were performed using one‐way ANOVA (**p < 0.01; ***p < 0.001; and ns, no significance).

Since FadD1 affects the QS gene expression levels in *P. aeruginosa*, we used electrophoretic mobility shift assays (EMSAs) to test whether the transcriptional regulation of the QS signal receptor genes and synthase‐encoding genes of *las*, *rhl*, and *pqs* QS systems is achieved through direct binding of FadD1 to the promoter. The 339‐, 321‐, 270‐, 330‐, 306‐, and 324‐bp DNA fragments of the *lasR*, *lasI*, *rhlR*, *rhlI*, *mvfR,* and *pqsA* promoters were used as probes. The EMSA results showed that the *lasR* promoter formed a stable DNA–protein complex with FadD1 (Figure 3A). The amount of the FadD1‐binding probe increased with the increase in the amount of FadD1 (Figure 3A). However, FadD1 did not bind to the other tested gene promoters, that is, *rhlR*, *mvfR*, *lasI*, *rhlI,* and *pqsA* (Figures S6 and S7).

In order to determine the DNA motifs recognized by FadD1 and genes directly regulated by FadD1, the chromatin immunoprecipitation sequencing (ChIP‐seq) analysis was performed. The result revealed the potential FadD1‐binding site as 5′‐AGGACGG‐3′ in the *lasR* gene promoter probe (Figure 3B). The results showed that FadD1 could directly bind to multiple target gene promoters to regulate their transcription and expression (Table S2). To verify this, three target genes, *flgM*, *katA,* and *PA0692*, were chosen for EMSA analysis. The results showed that, like *lasR*, the amounts of FadD1‐binding probes of *flgM*, *katA,* and *PA0692* were also increased with an increase in the amount of FadD1 (Figure S8). Then, the binding sites, 5′‐AGGACGG‐3′, 5′‐TGGCCGG‐3′, 5′‐AGGAGGG‐3′, and 5′‐AGGGCGG‐3′, were mutated from the *lasR*, *flgM*, *katA,* and *PA0692* promoter regions, respectively. The EMSA results showed that FadD1 could not form a DNA–protein complex with the mutated binding site promoters (Figures 3C and S9), indicating that this fragment is crucial for the binding of FadD1 to *lasR, flgM*, *katA*, and *PA0692* promoters. We then used RT‐qPCR to detect the changes of *lasR*, *katA*, *flgM*, and *PA0692* gene expression levels during different growth cycles (OD_600_ = 1.0, 2.0, and 3.0). The RT‐qPCR result indicated that the mutation of *fadD1* led to decreased expression levels in the *lasR*, *katA*, *flgM*, and *PA0692* (Figure S10). The degree of reduction varied among different genes, and the decreases were more pronounced in the later stages of growth (OD_600_ = 2.0 and 3.0). All the results indicated that FadD1 can bind to the promoters of multiple genes, but the regulatory activity on different genes varies, and it also varies in different growth cycles.

### The key to binding with DNA is the leucine zipper motif of FadD1

Since FadD1 can bind to *lasR* and regulate transcription, HMMER (profile Hidden Markov Models) was used to analyze the domain structure of FadD1. It was found that FadD1 has an AMP‐binding enzyme domain (AMP‐B) and an AMP‐binding enzyme C‐terminal domain (AMP‐B_C) (Figure 3D). We then purified the AMP‐B domain and the AMP‐B_C domain for EMSA (Figure 3E). The results showed that only the AMP‐B domain can bind to the *lasR* promoter region (Figures 3F,G). To determine the location of the DNA‐binding motif in the AMP‐B domain, we used the protein motif analysis website (https://www.novopro.cn/tools/motifscan.html). A structure called “leucine zipper” was characterized at 275 to 296 amino acids **L**TGNHNI**L**ITNPRD**L**PSMLKD**L** in the AMP‐B domain (Figures 3D and S11). The leucine zipper is composed of periodic repeats of leucine residues at every seven positions. This motif has been found in many eukaryotic transcription factors^45^. However, in the bacterial transcription factor, this motif is only found in the classical DNA‐binding HTH domain of the LysR family regulator MetR^46^ and a BDSF transcriptional regulator DsfR recently^47^. Therefore, we speculated that the four leucine residues L275, L282, L289, and L296 might be important for FadD1 binding to the target gene promoters. We then introduced four site‐specific substitutions into FadD1 (named FadD1^LM^) (Figure 3H), and found that the simultaneous mutation of L275A, L282A, L289A, and L296A abolished the binding of FadD1 to the *lasR* promoter (Figure 3I). In addition, *in trans* expression of FadD1^LM^ did not restore the biofilm formation, swarming motility, and pyocyanin production phenotypes of the ∆*fadD1* strain (Figure 3J–L). We then simulated the binding between FadD1 and the DNA‐binding site 5′‐AGGACGG‐3′ using the HDOCK server. The detailed binding sites of FadD1 and DNA are shown in Figure S12 and Table S3. The binding sites of FadD1 are in the leucine zipper structure. Therefore, the leucine zipper is the DNA‐binding motif of FadD1.

### FadD1 is a receptor of *cis*‐DA

**Figure 4:**
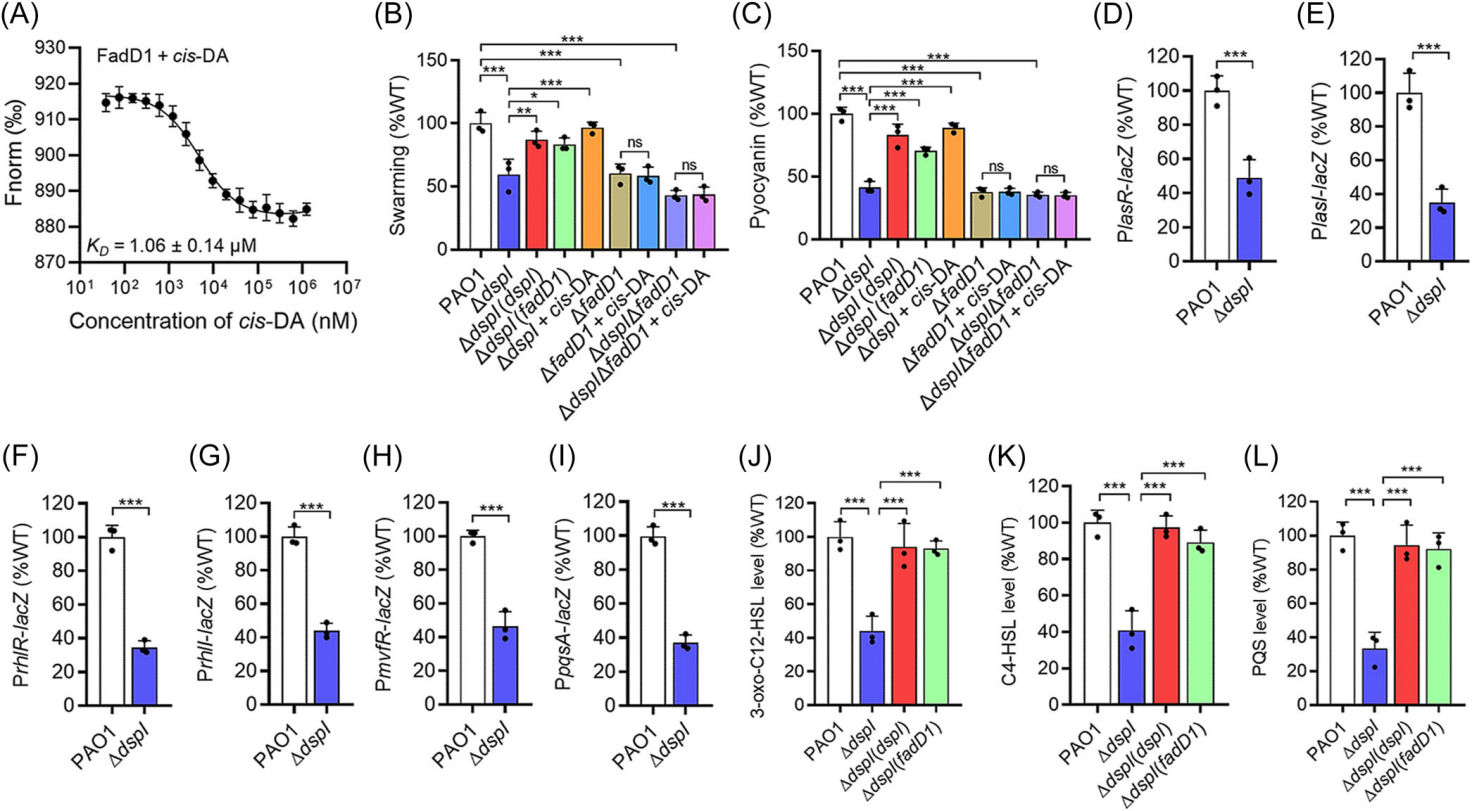
Effects of FadD1 on *cis*‐DA‐regulated phenotypes of *P. aeruginosa*. (A) Microscale Thermophoresis (MST) analysis of the binding of *cis*‐DA to FadD1. “Fnorm (‰)” indicates the fluorescence time trace changes in the MST response. (B, C) *In trans* expression of *fadD1* complemented swarming motility (B) and pyocyanin production (C) in the *dspI*‐deficient mutant complemented with fadD1. (D–L) Effects of *dspI* on the gene expression levels of *lasR* (D), *lasI* (E), *rhlR* (F), *rhlI* (G), *mvfR* (H), and *pqsA* (I), and the production of 3‐oxo‐C12‐HSL (J), C4‐HSL (K), and PQS (L) of the *P. aeruginosa* PAO1 strains. The production of QS signals in the *P. aeruginosa* PAO1 wild‐type strain was arbitrarily defined as 100%. The data are means ± standard deviations of three independent experiments. The statistical comparisons were performed using one‐way ANOVA (*p < 0.05; **p < 0.01; and ***p < 0.001).

Since *cis*‐DA is a DSF‐type QS signal in *P. aeruginosa*, and FadD1 acts as a global transcriptional regulator to regulate QS‐regulated phenotypes, we then examined whether there is an interaction between *cis*‐DA and FadD1. We purified the FadD1 protein for microscale thermophoresis (MST) analysis to determine whether FadD1 binds *cis*‐DA. As shown in Figure 4A, FadD1 exhibited binding activity to *cis*‐DA, and the estimated dissociation constant (*K*_*D*_) is 1.06 ± 0.14 μM. To further confirm that FadD1 is a receptor of *cis*‐DA, we generated the *cis*‐DA‐deficient mutant Δ*dspI*, the double‐deletion mutants ∆*dspI*∆*fadD1,* and *in trans* expressed *fadD1* in the Δ*dspI* and tested the motility and pyocyanin. The results demonstrated that the *in trans* expression of *fadD1* restored the motility and pyocyanin phenotypes of Δ*dspI* (Figure 4B,C). We also observed that exogenous addition of *cis*‐DA restored the motility and pyocyanin of ∆*dspI* (Figure 4B,C); however, there was no effect on ∆*fadD1* and ∆*dspI*∆*fadD1* (Figure 4B,C). We then detected the *lasR*, *lasI*, *rhlR*, *rhlI*, *mvfR,* and *pqsA* gene expression levels in the wild‐type and Δ*dspI* strains. The results showed that the gene expression levels of *las*, *rhl*, and *pqs* QS system genes were significantly reduced in the Δ*dspI* strain (Figure 4D–I). Therefore, we measured and compared the production of QS signals (3‐oxo‐C12‐HSL, C4‐HSL, and PQS) in the wild‐type, ∆*dspI*, and ∆*dspI*(*dspI*) strains. The result indicated that the production of these three QS signals decreased in the ∆*dspI* strain (Figure 4J–L). These results indicated that both *cis*‐DA and FadD1 positively regulate the *lasR* transcription in *P. aeruginosa*, thereby regulating the *las*, *rhl*, and *pqs* QS systems by the usual QS regulatory circuits.

### The binding ability of FadD1 to its target gene promoters is enhanced by *cis*‐DA

**Figure 5:**
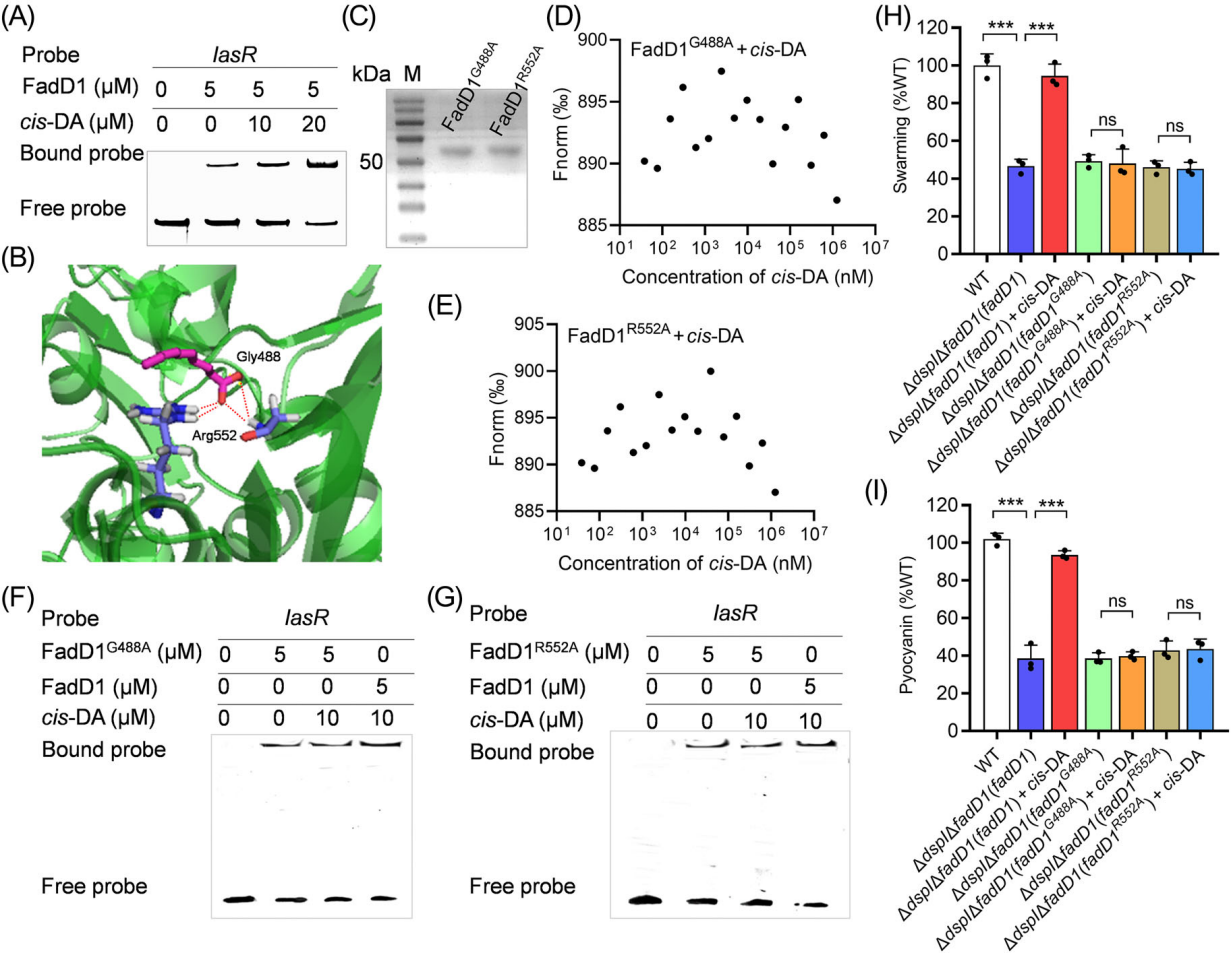
Effects of *cis*‐DA on the binding of FadD1 to the promoters of the target genes. (A) EMSA analysis of the in vitro binding of FadD1 to the promoter of *lasR* with the addition of different amounts of *cis*‐DA. (B) Potential residues (shown as stick models and labeled) of FadD1 involved in *cis*‐DA binding analyzed by AutoDocking software. The hydrogen bonds are represented by red dashed lines. (C) SDS‒PAGE of purified FadD1 variants. (D, E) MST analysis of the binding of BDSF to the FadD1^G488A^ (D) and FadD1^R552A^ (E). (F, G) EMSA analysis of the in vitro binding of FadD1^G488A^ (F) and FadD1^R552A^ (G) to the promoters of *lasR* with the addition of different amounts of *cis*‐DA. The EMSA experiments were performed three times, and representative images from one experiment are shown. (H, I) Analysis of the sensing of *cis*‐DA by FadD1^G488A^ and FadD1^R552A^. Swarming motility (H) and pyocyanin production (I) regulated by *cis*‐DA in *P. aeruginosa* are shown. The data are means ± standard deviations of three independent experiments. The statistical comparisons were performed using one‐way ANOVA (***p < 0.001; ns, no significance).

To determine how *cis*‐DA affects the regulatory activity of FadD1, we used EMSA analysis to examine the effect of *cis*‐DA on FadD1 binding to the *lasR* promoter. The results showed that the addition of *cis*‐DA enhanced the binding ability of FadD1 to the *lasR* promoter, and the amount of probe bound to FadD1 increased with an increase of the *cis*‐DA concentration (Figure 5A).

Then, we attempted to use Autodocking analysis to identify the *cis*‐DA‐binding sites in FadD1. The results showed two amino acid residues, Gly488 (G488) and Arg552 (R552), which might be critical for FadD1 binding to *cis*‐DA (Figure 5B). To determine whether these two amino acid residues are associated with binding to *cis*‐DA, we subsequently constructed two single‐point mutants (FadD1^G488A^ and FadD1^R552A^) (Figure 5C) for MST analysis. It was found that the mutant proteins, FadD1^G488A^ and FadD1^R552A^, did not bind to *cis*‐DA (Figure 5D,E). Consistent with this, EMSA analysis showed that the *cis*‐DA‐binding site (Gly488 and Arg552) mutants lost the influence of *cis*‐DA on FadD1 (Figure 5F,G). Moreover, *in trans* expression of *fadD1* ^*G488A*^ or *fadD1* ^*R552A*^ could not restore the motility and pyocyanin (Figure 5H,I), and exogenous addition of 20 μM *cis*‐DA showed no effect on the ∆*dspI*∆*fadD1*(*fadD1* ^*G488A*^) and ∆*dspI*∆*fadD1*(*fadD1* ^*R552A*^) (Figure 5H,I). Thus, we concluded that FadD1 is a specific transcriptional regulator responsive to *cis*‐DA in *P. aeruginosa*.

### Leucine zipper motif plays a key role in regulating activity of FadD1

Our study found that the DNA‐binding motif of FadD1 is a leucine zipper motif. Therefore, we used the protein motif analysis website (https://www.novopro.cn/tools/motifscan.html) to analyze whether the other FadD protein, FadD2, contain a leucine zipper structure, and found that FadD2 did not contain a leucine zipper structure. EMSA analysis showed that FadD2, which shares about 59.82% amino acid identity with FadD1, did not bind to the *lasR* gene promoter (Figure S13A,B). To further investigate the role of the FadD1 leucine zipper motif, we reconstructed FadD2 by adding the leucine zipper motif to the same part of FadD2 (named FadD2_LZ_) and tested whether the reconstructed FadD2 has regulatory activity similar to that of FadD1. Therefore, FadD2_LZ_ was purified to homogeneity (Figure S13C), and we carried out an assay to determine whether it could bind to the *lasR* promoter. Intriguingly, we found that, like FadD1, the *lasR* promoter formed stable DNA–protein complexes with FadD2_LZ_ and led to a slower migration rate. The amount of probe binding to FadD2_LZ_ increased with an increase in the amount of FadD2_LZ_ (Figure S13D). Interestingly, the addition of exogenous *cis*‐DA also enhanced the binding of FadD2_LZ_ to the *lasR* promoter at 20 µM (Figure S13E). In addition, *in trans* expression of the *fadD2*_*LZ*_ gene rescued the defective swarming motility and pyocyanin production phenotypes of the Δ*fadD1* strain (Figure S13F,G). We also found that adding exogenous *cis*‐DA can restore the motility and pyocyanin of ∆*dspI*∆*fadD1*(*fadD2*_*LZ*_) (Figure S13F,G). These results suggested that the leucine zipper motif plays a critical role in the regulatory activity of FadD1.

### FadD1 is mainly used as a QS regulator rather than a long‐chain fatty acid‐CoA ligase

Many studies have shown that fatty acid‐CoA ligase plays a key role in fatty acid metabolism by catalyzing fatty acid activation^35^, ^36^. To test whether FadD1 plays a role in the fatty acid metabolism, we measured the enzymatic activity in vitro and found that FadD1 showed high activity on lauric acid (C12:0), while it displayed moderate activity on BDSF but no activity on *cis*‐DA (Figure S14A). In addition, the previous study found that, in *Escherichia coli*, mutations in the substrate‐binding sites (Trp433, Thr436, and Arg453) lead to complete inactivation of fatty acid‐CoA synthetase^37^. We subsequently constructed three single‐point mutants (FadD1^W434A^, FadD1^T437A^, and FadD1^R454A^) to verify whether these mutations affect the enzymatic activity of FadD1 (Figure S14B). Similar to the previous studies, FadD1^W434A^, FadD1^T437A^, and FadD1^R454A^ showed no enzyme activity on *cis*‐DA, BDSF, and lauric acid (C12:0) (Figure S14C–E). To clarify the relationship between the FadD1 enzyme activity and perception of *cis*‐DA, we chose the mutant FadD1^R454A^ to test its regulatory activity. We *in trans* expressed *fadD1* ^*R454A*^ in the ∆*dspI*∆*fadD1* mutant strain. Interestingly, it was found that adding exogenous *cis*‐DA could restore the motility of ∆*dspI*∆*fadD1*(*fadD1* ^*R454A*^) (Figure S14F). Consistent with this, EMSA analysis showed that mutation of the substrate‐binding site (Arg454) did not affect the perception of *cis*‐DA (Figure S14G). We also detected competitive changes between the *B. cenocepacia* H111 strain and *P. aeruginosa* wild‐type, ∆*fadD1*, ∆*fadD1*(*fadD1*), ∆*fadD1*(*fadD1* ^*LM*^), and ∆*fadD1*(*fadD1* ^*R454A*^) through CFU counting. The result also showed that the CFUs of the *P. aeruginosa* ∆*fadD1* and ∆*fadD1*(*fadD1* ^*LM*^) strains were much lower than those of the *P. aeruginosa* wild‐type and ∆*fadD1*(*fadD1*) strains when they were cocultured with the *B. cenocepacia* H111 (Figure S15A). Also, the CFUs of the *B. cenocepacia* H111 strain cocultured with *P. aeruginosa* ∆*fadD1* and ∆*fadD1*(*fadD1* ^*LM*^) strains were higher than that of the *B. cenocepacia* H111 strain cocultured with *P. aeruginosa* wild‐type and ∆*fadD1*(*fadD1*) strains (Figure S15B). These results indicated that replenishing the *fadD1* ^*LM*^ gene cannot restore the competitiveness of the *P. aeruginosa* ∆*fadD1* strain. At the same time, we found that ∆*fadD1*(*fadD1* ^*R454A*^) could partially restore the competitiveness of the *P. aeruginosa* ∆*fadD1* strain (Figure S15). All these results indicated that the role of *fadD1* in the competition of *P. aeruginosa* is mainly related to its function as a QS regulator, rather than its ability to degrade BDSF.

Although FadD1 showed degradation activity on BDSF, we found that FadD1 tightly binds to BDSF with an estimated dissociation constant (*K*_*D*_) of 1.65 ± 0.21 μM (Figure S16A). Interestingly, the addition of exogenous BDSF also enhanced the binding of FadD1 to the *lasR* promoter probe at a final concentration of 20 µM (Figure S16B). To distinguish the role of BDSF as a “substrate” or “ligand” of FadD1, we then performed EMSA analysis, and the result showed that mutation of the *cis*‐DA‐binding sites (Arg552) did not affect BDSF on FadD1 (Figure S16C). However, mutation of the substrate‐binding site (Arg454) completely abolished the effect of BDSF on the binding of FadD1 to the target gene promoter (Figure S16D). We then examined the transcriptional expression levels of P*lasR*‐*lacZ* in the *P. aeruginosa* wild‐type, ∆*dspI*∆*fadD1*, ∆*dspI*∆*fadD1*(*fadD1*), ∆*dspI*∆*fadD1*(*fadD1* ^*LM*^), and ∆*dspI*∆*fadD1*(*fadD1* ^*R454A*^) strains with or without BDSF and *cis*‐DA signals (5, 25, 100, and 250 μM). The result indicated that the low concentrations of BDSF and *cis*‐DA signals (5 and 25 μM) positively regulated the expression of *lasR*, while the high concentrations of BDSF and *cis*‐DA signals (100 and 250 μM) inhibited the expression of *lasR* (Figure S17), which is consistent with a previous report^50^. Further research is still required to identify the specific mechanism. The result also showed that *in trans* expression of the *fadD1* ^*LM*^ gene in the ∆*dspI*∆*fadD1* strain could not restore the expression of *lasR*, regardless of whether BDSF and *cis*‐DA signals were added or not. However, *in trans* expression of *fadD1* or *fadD1* ^*R454A*^ in the ∆*dspI*∆*fadD1* strain could partially restore the expression of *lasR*. Also, exogenous addition of low concentrations of the *cis*‐DA signal (5 and 25 μM) could almost restore the expression level of *lasR* in ∆*dspI*∆*fadD1*(*fadD1*) and ∆*dspI*∆*fadD1*(*fadD1* ^*R454A*^). However, exogenous addition of low concentrations of the BDSF signal (5 and 25 μM) could almost restore the expression level of *lasR* in the ∆*dspI*∆*fadD1*(*fadD1*), but could not restore ∆*dspI*∆*fadD1*(*fadD1* ^*R454A*^) (Figure S17). All these results further demonstrated that mutation of the substrate‐binding site (Arg454) completely eliminated the effect of BDSF on the FadD1 regulatory activity, while it did not affect the action of *cis*‐DA on FadD1, and indicated that *cis*‐DA, as a signal, affects the regulatory activity of FadD1, while BDSF, as a substrate, can also affect the regulatory activity of FadD1.

### Homologs of FadD1 are widespread in bacteria

To determine whether FadD1 is widely distributed, the BLAST program was used to search for the homologs of FadD1 in the genome database. It was indicated that homologs of FadD1 are distributed across many different bacterial species, including *Pseudomonas*, *Azotobacter*, *Azomonas*, and *Halopseudomonas* (Table S4). Furthermore, the homologs of FadD1 in the genera *Pseudomonas, Azotobacter,* and *Azomonas* contain a leucine zipper structure (Table S4). We then selected *P. fluorescens* Migula ATCC17518, which contains both a FadD1_Pf_ protein with a leucine zipper structure and a *lasR*_*Pf*_ gene, and the FadD1_Pf_ protein was purified by affinity chromatography and tested for its interaction with the *lasR*_*Pf*_ gene promoter. The EMSA results showed that the FadD1_Pf_ protein from *P. fluorescens* could also bind to the *lasR*_*Pf*_ gene promoter (Figure S18), indicating that the transcriptional regulatory mechanism of FadD1 might be widely conserved in bacteria.

## DISCUSSION

Fatty acid‐CoA ligase plays a key role in fatty acid metabolism, as it can catalyze the activation of fatty acids into fatty acyl‐CoA in bacteria^37^. In our research, we found that the fatty acid‐CoA ligase FadD1 of *P. aeruginosa* not only shows high activity for the catalysis of lauric acid to lauric acid‐CoA (Figure S14A), and plays a vital role in bacterial competition with *B. cenocepacia* by degrading its QS signal BDSF (Figure 1), but also plays a critical role in the regulation of *P. aeruginosa* physiology and pathogenesis by acting as a receptor of its QS signal *cis*‐DA (Figure 2 and Table S1). We demonstrated that FadD1 can directly bind to the target gene promoters, and thereby regulate the transcription levels of the target genes (Figures 3A and S8). It was further identified that deleting the leucine zipper structure eliminated the binding of FadD1 to the promoter of the target genes (Figure 3I). Interestingly, we found that FadD2 does not contain a leucine zipper structure, and EMSA analysis indicated that FadD2 did not bind to the *lasR* gene promoter (Figure S13B). However, the reconstructed FadD2 with the addition of the FadD1 leucine zipper structure to the same part of FadD2 (named FadD2_LZ_) showed regulatory activity similar to that of FadD1. It could bind to the *lasR* gene promoter and restore the *fadD1* mutant phenotypes (Figure S13). Furthermore, our findings indicate that FadD1 is widely present and conserved in many bacterial species, including *Pseudomonas*, *Azotobacter*, and *Azomonas* species (Table S4), indicating that FadD1 with a leucine zipper structure might be a new family of transcriptional regulator proteins widely conserved in bacteria.

The fatty acid molecule *cis*‐DA was first identified in *P. aeruginosa* to serve as the autoinducer of biofilm dispersion^31^. It has also been found to induce biofilm dispersion in many Gram‐negative and Gram‐positive bacteria, as well as in the fungal pathogen *Candida albicans* ^31^. Although the previous study reported that the DspS (PA4112, PA14_10770), a two‐component sensor/response regulator hybrid protein, is crucial for the response of intercellular signaling molecule *cis*‐DA and is essential for native biofilm dispersion^34^, there was no exploration of other functions of *cis*‐DA. In this study, we found that deletion of *dspI*, which is the synthase of *cis*‐DA, caused an impairment in motility and pyocyanin production (Figure 4B,C). Interestingly, in *trans* expression of *fadD1* restored the motility and pyocyanin phenotypes of the ∆*dspI* strain (Figure 4B,C). However, the addition of exogenous *cis*‐DA did not affect the phenotypes of ∆*fadD1* and ∆*dspI*∆*fadD1* (Figure 4B,C). Moreover, *cis*‐DA is shown to bind to FadD1 with high affinity and enhance the binding activity of FadD1 to the promoters of the target gene (Figures 4A and 5A). We also found that the two amino acid residues of Gly488 (G488) and Arg552 (R552) play important roles in the interaction between FadD1 and *cis*‐DA (Figure 5). Mutations at Gly488 and Arg552 abolished the binding between FadD1 and *cis*‐DA (Figure 5D,E). Another interesting finding is that the mutation of the substrate‐binding site Arg454 (R454) completely abolished the enzyme activity of FadD1 on both BDSF and lauric acid (C12:0) (Figure S14C–E), but did not affect the effect of *cis*‐DA on FadD1 (Figure S14G), indicating that the *cis*‐DA‐binding site is different from the substrate‐binding site in FadD1. In summary, FadD1 acts as a new transcriptional regulator of *cis*‐DA QS signaling.

BDSF has been identified to be a DSF‐family QS signal in *B. cenocepacia* ^11^. As *cis*‐DA shares a similar chemical structure to that of BDSF, we then continued to explore the interaction between BDSF and FadD1. The results showed that FadD1 tightly binds to BDSF with an estimated dissociation constant (Figure S16A), and the addition of exogenous BDSF also enhanced the binding of FadD1 to the *lasR* promoter (Figure S16B). The EMSA analysis showed that the mutation of *cis*‐DA‐binding sites abolished the perception of *cis*‐DA (Figure 5), but did not affect the action of BDSF (Figure S16C). However, EMSA analysis also indicated that mutation of the substrate‐binding site (Arg454) completely abolished the effect of BDSF on the binding of FadD1 to the promoter of target genes (Figure S16D), while it did not affect the effect of *cis*‐DA on FadD1 (Figure S14G). We also found that *in trans* expression of the *fadD1* ^*LM*^ gene in the ∆*dspI*∆*fadD1* strain could not restore the expression of *lasR*, regardless of whether the BDSF signal was added or not. However, *in trans* expression of *fadD1* and the *fadD1* ^*R454A*^ gene in the ∆*dspI*∆*fadD1* strain could partially restore the expression of *lasR*. While exogenous addition of a low concentration of the BDSF signal (5 and 25 μM) could almost restore the expression level of *lasR* in the ∆*dspI*∆*fadD1*(*fadD1*), it could not restore ∆*dspI*∆*fadD1*(*fadD1* ^*R454A*^) (Figure S17). All these results suggested that BDSF also influences the binding of FadD1 to the target gene promoters in vivo as a substrate of FadD1.

**Figure 6:**
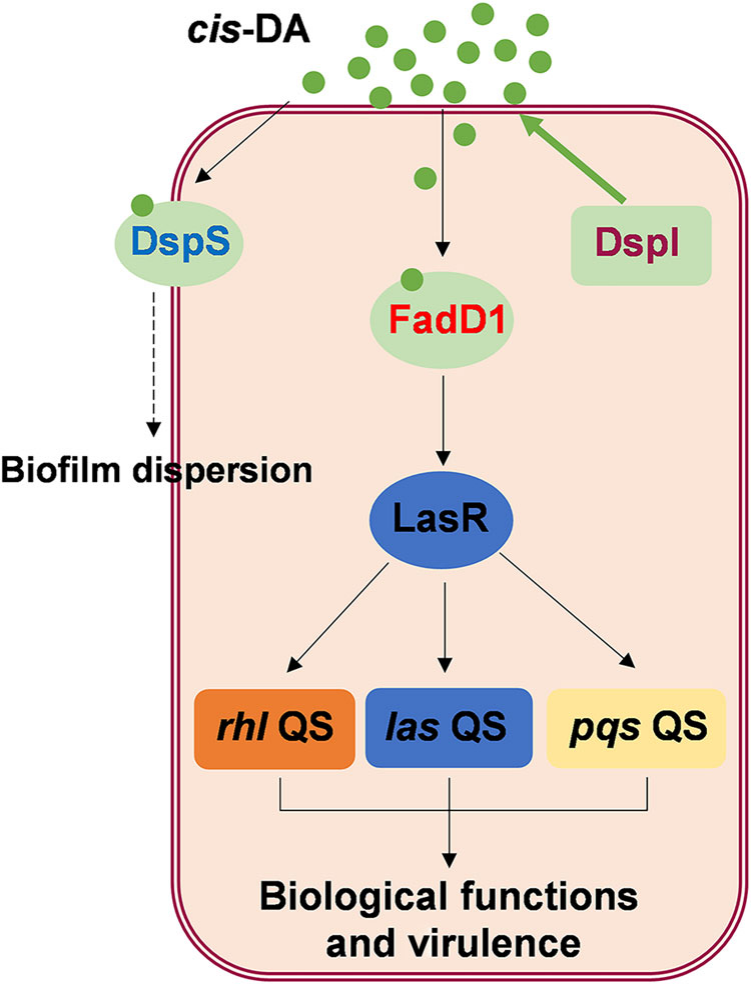
Schematic representation of *cis*‐DA‐regulated QS signaling in *P. aeruginosa*. DspI synthesizes the *cis*‐DA signal molecule. FadD1, the signal receptor of *cis*‐DA, perceives the *cis*‐DA signal and regulates the transcription of *lasR*, which positively regulates the *las*, *rhl,* and *pqs* QS systems in *P. aeruginosa*. DspS is also the sensor of *cis*‐DA to be involved in biofilm dispersion in response to *cis*‐DA. The individual QS circuits are highly interconnected.

There are at least three QS systems in *P. aeruginosa*, namely, *las*, *rhl,* and *pqs*, which are involved in the regulation of a wide range of genes important for metabolism and virulence^18^, ^19^. These QS systems are hierarchically interrelated, and the *las* system was verified to control both the *rhl* and *pqs* systems^17^, ^30^. In this study, we found that FadD1 affects the QS‐regulated phenotypes, including biofilm formation, swarming, and pyocyanin in *P. aeruginosa* (Figure 2A–C). Surprisingly, we also identified that FadD1 is a *cis*‐DA receptor protein in *P. aeruginosa* that positively regulates the transcriptional expression of *lasR*, thereby regulating the *las*, *rhl,* and *pqs* QS systems (Figures 2 and 6). Previous studies have shown that Vfr, PA3699, PsdR, and PsrA can regulate the expression of *lasR* in *P. aeruginosa* ^54^, ^55^, ^56^, ^57^, ^58^. Some studies have also found that RsaL coordinates the sequential biosynthesis of AHL QS signals, representing advanced participation at the top of the hierarchical structure^59^. These results indicated that FadD1 is not only a response transcriptional regulator of *cis*‐DA but also an important upstream regulator of the QS signaling network in *P. aeruginosa*. FadD1 regulates numerous target genes and physiological functions by modulating the QS signaling systems, which broadens our understanding of the QS signaling network of *P. aeruginosa*.

In summary, our findings suggest that FadD1, which contains a leucine zipper structure in the AMP‐B domain, is not only an enzyme of fatty oxidation but also a receptor of *cis*‐DA that directly controls the expression of target genes and regulates the QS network in *P. aeruginosa*. In addition, the BLAST search revealed that homologs of FadD1 containing the leucine zipper structure are highly conserved in a range of bacteria (Table S4), indicating that the regulatory mechanism of FadD1 might exist in various bacterial species. Consistently, our recent study revealed that the fatty acyl‐CoA ligase DsfR (BCAM2136) in *B*. *cenocepacia* H111, which also contains the leucine zipper motif, controls the virulence by sensing BDSF as a global transcriptional regulator^47^. Different from FadD1, DsfR has a specific BDSF‐sensing site and showed no enzyme activity on BDSF, while FadD1 only binds with BDSF on the substrate‐binding sites. These results indicate that FadD1 and DsfR share some common characteristics and are distinguished from each other. In conclusion, our work presents a unique and widely conserved signal receptor of *cis*‐DA, which might be an important new type of QS signal receptor in bacteria.

## MATERIALS AND METHODS

### Bacterial strains and culture conditions

The bacterial strains and plasmids used in this study are listed in Table S5. *P. aeruginosa* PAO1, *P. fluorescens* Migula ATCC17518, and *B. cenocepacia* H111 strains from the American Type Culture Collection (ATCC) were maintained in Luria–Bertani broth (LB) at 37°C. The antibiotic concentrations used were as follows: ampicillin and kanamycin 100 µg ml^−1^, tetracycline 10 µg ml^−1^, and gentamicin 10 µg ml^−1^. BDSF and *cis*‐DA were dissolved in methanol and added to the culture medium.

### BSDF extraction

The extraction of BDSF from culture supernatants was carried out following previously described methods^11^, ^47^, ^60^. To quantify BDSF production of *B. cenocepacia*, 100 ml of the supernatant was collected. Its crude ethyl acetate extract was filtered through a 0.22 µm Minisart filter unit and condensed to 1 ml for LC‐MS analysis.

### Quantification of BDSF and *cis*‐DA

Quantification of BDSF and *cis*‐DA was performed by ultrahigh‐performance liquid chromatography‐electrospray ionization tandem mass spectrometry (UHPLC‐ESI‐MS/MS) using a Waters C18 column (1.8 µm, 150 × 2.1 mm) as previously described^47^, ^60^.

### Protein expression and purification

The coding regions of proteins were attached and fused to the pET‐21a, pET‐28a, and PDBHT2 expression vectors, and transformed into *E. coli* strain BL21. The Ni‐NTA resin was used for affinity purification of the fusion proteins. The His‐tag was removed using TEV protease (Beyotime), and the cleaved fusion protein was analyzed by sodium dodecyl sulfate – polyacrylamide gel electrophoresis (SDS‐PAGE)^47^, ^61^, ^62^.

### Enzyme activity assays

The enzyme activity was measured according to the previously described method^47^, ^63^. *cis*‐DA, BDSF, or lauric acid (C12:0) was dissolved in TME buffer (200 µM) and evenly mixed with FadD1 (20 µM) for incubation at 37°C. The reaction was stopped by immersing in a boiling water bath at 5, 15, 30, and 60 min. *cis*‐DA, BDSF, or lauric acid (C12:0) levels were determined by LC‐MS spectrometry.

### Construction of in‐frame deletion mutants and complementation

*P. aeruginosa* PAO1 was used for the generation of an in‐frame deletion mutant of *fadD1* and *dspI* using the method described previously^14^. The primers used are listed in Table S6. For complementation analysis, the coding regions of *fadD1* and *dspI* were cloned into the plasmid pBBR1‐MCS5, pBBR1‐MCS2, and pLAFR3. The constructed recombinant plasmids were electroporated into the *P. aeruginosa* PAO1 mutant^14^, ^61^.

### Bacterial growth analysis

Overnight bacterial culture was inoculated into fresh LB media to an OD_600_ of 0.01. A 10 ml cell suspension was grown at 37°C with shaking at 220 rpm, and the optical density was determined at 600 nm every 4 h^47^.

### Competition assay in the mixed culture

The green and mCherry fluorescent protein expression vectors were introduced into *P. aeruginosa* PAO1 WT, Δ*fadD1,* and Δ*fadD1*(*fadD1*) strains and the *B. cenocepacia* H111 strain by electroporation. Then, the *P. aeruginosa* PAO1 WT, Δ*fadD1,* and Δ*fadD1*(*fadD1*) strains were cocultured with the *B. cenocepacia* H111 strain at an initial ratio of 1:4 (vol/vol) at OD_600_ = 0.1 for 12 h. The quantification of BDSF was performed using an LC‐MS system, and the green or mCherry fluorescence values were determined using Agilent BioTek SH1M‐SN^64^.

### *P. aeruginosa* phenotype assay

For the analysis of biofilm formation^47^, ^65^, overnight bacterial cells were inoculated into LB media (150 μl per well) in 96‐well polypropylene microtiter plates. Microtiter plates with fitted lids were incubated at 37°C for 12 h. The plates were then washed and stained for 20 min with 1% (weight/vol) crystal violet. Then, the plates were washed three times, and 200 µl of 95% (vol/vol) ethanol was added to release the biofilm. Biofilm was determined by measuring the absorbance of 570 nm.

Motility was determined on 0.3% semisolid agar (Becton, Dickinson and Company). Bacteria were inoculated into the center of plates containing 0.8% tryptone, 0.5% glucose, and 0.3% agar. The plates were incubated at 30°C for 18 h, and then the diameter of the colony was measured^61^.

The pyocyanin assay was performed using the previously described method^50^. Bacteria were cultured for approximately 12 h at 37°C until the solution turned significantly green and were then collected by centrifugation. Double‐volume chloroform was added, and vigorous shaking was carried out for 30 min. Then, the solvent phase was transferred, and 1 ml of 1 mol/l HCl was added and mixed gently to transfer pyocyanin to the aqueous phase to measure the absorbance at 520 nm.

### *B. cenocepacia* phenotype assay

Biofilm formation of *B. cenocepacia* was basically determined as previously described for *P. aeruginosa*. Swarming motility was determined by measuring the diameter of the colony on semisolid agar (0.8% tryptone, 0.5% glucose, and 0.3% aga) according to the previously described method. Protease assay was carried out as previously described^14^. *B. cenocepacia* bacteria were cultured to the logarithmic growth phase. *B. cenocepacia* strains were cultured for approximately 12 h at 37°C and the supernatants were collected. One hundred microliters of the supernatant was incubated with equal volume of azocasein (5 mg/ml) for 30 min at 30°C and 400 μl of 10% (weight/vol) TCA buffer was added to stop the reaction. After 2 min, centrifugation was performed at 13,000 rpm for 1 min, and the supernatants were removed and mixed with 700 μl of 525 mM NaOH. The protease activity was determined by measuring the azopeptide supernatant absorbance at 442 nm.

### Cytotoxicity assay

The release of LDH from human A549 cells was measured using a cytotoxicity detection kit (Roche) to assess the cytotoxicity of *P. aeruginosa* strains, as previously described^61^. A549 cells were cultured in DMEM at 37°C with 5% CO_2_, added to 96‐well plates (5 × 10^4^ cells per well), and grown to ~90% confluence. Fresh *P. aeruginosa* cells (OD_600_ = 1.0) were washed and diluted in DMEM. Then, the diluted bacterial cells were seeded into A549 cells with a multiplicity of infection (MOI) of approximately 1000. After incubation for 8 h, the cytotoxicity was determined by measuring the LDH released in the supernatants.

### Construction of the promoter‐*lacZ* fusion reporter strain and measurement of β‐galactosidase activity

The target gene promoters were amplified using the reporter primers listed in Table S6. Then, the promoters were inserted into the upstream of the *lacZ* gene without the promoter in the pME2‐*lacZ* vector. Measurement of β‐galactosidase activities was carried out according to the previously described method^14^, ^47^, ^61^, ^62^.

### RT‐qPCR assay

*P. aeruginosa* strains were cultured to the logarithmic growth phase and collected for total RNA isolation using the Eastep Super Total RNA Extraction Kit (Promega). The HiScript III 1st Strand cDNA Synthesis Kit (Yeasen) was used for cDNA synthesis. RT‐qPCR was performed on the QuantStudio 7 RT‐qPCR System (Thermo Fisher Scientific). The target gene expression was normalized to the 16S RNA expression level. The relative transcript abundance was calculated using the $2^{‐\Delta \Delta C_{t}}$ method^47^, ^62^.

### Quantitative analysis of QS signal production in *P. aeruginosa*

The production of QS signals in *P. aeruginosa* was assessed by UHPLC‐ESI‐MS/MS using a Waters C18 column (1.8 µm, 150 × 2.1 mm) as previously described^53^. 0.1% formic acid/water and acetonitrile were used as mobile phases for detection in positive ion mode.

### RNA‐Seq analysis

The RNA of *P. aeruginosa* strains (OD_600_ = 1.0) was isolated using the Eastep Super Total RNA Extraction Kit (Promega). The RNA‐Seq analysis was performed by the Wuhan IGENEBOOK Biotechnology Co. Ltd., as previously described^66^, ^67^.

### EMSA

EMSAs were conducted as previously described using the Thermo Scientific kit, with minor modifications (Thermo Fisher Scientific)^47^, ^53^, ^60^, ^61^. The DNA probes were prepared by PCR amplification and 3‐end‐labeled with biotin following the manufacturer's instructions. Then, the labeled probe was combined with protein in the binding buffer at room temperature for 20 min, and separated by the 5% polyacrylamide gel. The labeled probes were detected using the Biotin luminescent detection kit after UV cross‐linking.

### ChIP‐Seq analysis

ChIP‐Seq was performed by the Wuhan IGENEBOOK Biotechnology Co. Ltd., as previously described, with some modifications^68^. The immunoprecipitation was performed using the anti‐HIS antibody (ab9108, 1:1000, Abcam), and ChIP‐enriched DNA fragments were sequenced on Illumina HiSeq. 2000^47^, ^69^.

### Molecular docking of FadD1

The 3D structure of DNA was constructed in MOE1 DNA/RNA Builder. Protein–DNA docking in the HDOCK2 server was used for the FadD1 and DNA analyses as previously described^70^, ^71^. The FadD1 (Q9HYU4)3D structure from AlphaFold2 was set as the receptor, and the DNA 3D structure was set as the ligand^47^, ^72^. The docking and binding site analyses were performed using AutoDock and PyMOL. AutoDock software was used to dock the crystal structure of FadD1 from the AlphaFold2 with *cis*‐DA. The ligand conformation with the highest score was visualized in 3D by PyMOL and 2D by Protein Plus to identify the potential binding sites.

### Microscale thermophoresis assay

Protein‐binding experiments were conducted on labeled protein and *cis*‐DA or BDSF using the Nano Temper 16 Monolith NT.115 instrument as previously described^47^, ^62^. The different concentrations of *cis*‐DA or BDSF and labeled protein were mixed and added to the capillaries to measure fluorescence.

### Statistical analysis

The data are presented as means ± standard deviations. Statistical analyses were performed using Prism 8 software (GraphPad). Statistical significance is indicated as follows: *p < 0.05; **p < 0.01; ***p < 0.001; and ns, no significance (one‐way ANOVA or two‐way ANOVA). All experiments were repeated at least three times.

## AUTHOR CONTRIBUTIONS

**Shihao Song**: Conceptualization, data curation, formal analysis, funding acquisition, investigation, methodology, project administration, resources, software, supervision, validation, visualization, writing—original draft, writing—review and editing. **Jingyun Liu**: Data curation, formal analysis, investigation. **Bing Wang**: Data curation, formal analysis, investigation. **Yang Si**: Data curation, investigation. **Hongguang Han**: Data curation, investigation. **Xiuyun Sun**: Data curation, investigation. **Mingfang Wang**: Data curation, investigation. **Binbin Cui**: Data curation, investigation. **Guangliang Wu**: Formal analysis. **Yongliang Huo**: Formal analysis. **Liangxiong Xu**: Formal analysis. **Beile Gao**: Formal analysis. **Liang Yang**: Formal analysis. **Xiaoxue Wang**: Formal analysis. **Lian‐Hui Zhang**: Formal analysis. **Yinyue Deng**: Conceptualization, formal analysis, funding acquisition, supervision, writing—original draft, writing—review and editing.

## ETHICS STATEMENT

No animals were used in this study.

## CONFLICT OF INTERESTS

The authors declare no conflict of interests.

## Supporting information

SI‐Song‐mLife.

## Data Availability

RNA‐seq and ChIP‐seq data generated in this article have been deposited in the National Center for Biotechnology Information Sequence Read Archive (SRA) database under accession code PRJNA1121197 (https://www.ncbi.nlm.nih.gov/bioproject/PRJNA1121197) and PRJNA1120965 (https://www.ncbi.nlm.nih.gov/bioproject/PRJNA1120965), respectively. All relevant data are within the paper and its Supporting Information files.
